# StudyPortal – Geovisualization of Study Research Networks

**DOI:** 10.1007/s10916-019-1493-0

**Published:** 2019-12-10

**Authors:** Julian Varghese, Michael Fujarski, Martin Dugas

**Affiliations:** Institute of Medical Informatics, University of Münster, Albert-Schweitzer-Campus 1, Gebäude A11, 48149 Münster, Germany

**Keywords:** Clinical trials, Registries, Geographic information systems, UMLS, MEDLINE

## Abstract

StudyPortal was implemented as the first multilingual search platform for geographic visualization of clinical trials and scientific articles. The platform queries information from ClinicalTrials.gov, PubMed, a geodatabase and geographic maps to enable geospatial study search and real-time rendering of study locations or research networks on a map. Thus, disease-specific clinical studies or whole research networks can be shown in a geographic proximity. Moreover, a semantic layer enables multilingual disease input and autosuggestion of medical terms based on the Unified Medical Language System. The portal is accessible on https://studyportal.uni-muenster.de. This paper presents details on implementation of the novel search platform, its search evaluation and future work.

## Introduction

International trial databases as ClinicalTrials.gov provide powerful search platforms to study the current clinical research landscape [1–3]. The importance of searching trial databases has recently increased, particularly after 2005, as several initiatives for study registration have been implemented [4, 5]. A lack of transparency and consistency has been reported for some study areas and types [6]. However, a systematic analysis focusing on large randomized controlled trials and new drugs shows a trend towards comprehensive international study registration at Clinicaltrials.gov [7]. Sensitivity and precision were higher for those trials, than searching via other trial registries such as the European Clinical Trial Register and WHO-based International Meta-Registry, even for non-US trials [7]. Using the search platform on ClinicalTrials.gov, a user can select a number of search fields and will gain access to a list of registered trials that meet the search criteria. Each listed trial provides information, as for example, the study design, its therapeutic area or medical condition, sponsors, principal investigators and more importantly for the scope of this work: the study site locations. By linking these locations to geographic coordinates and further processing, we unlock two use cases for clinical research and patient care, which are highly relevant but yet unexploited:

First, health care providers and their patients suffering from cancer or chronic diseases could access an overview of suitable clinical trials with potentially new suitable treatment options, close to the patient’s place of residence or patient’s preferred location.

Second, clinical researchers could generate a map-based overview of clinical research networks that have conducted similar research and therefore could synergistically share expertise. This is particularly useful when new research networks are being formed or extended and suitable research partners need to be identified.

Currently, both of the presented use cases are only realizable by several tedious manual searches. In addition, more advanced location queries, as for instance: “Find the nearest clinical studies within a given distance of 200 km from a specific location” are not executable, since geospatial relations (GPS coordinates, longitude and latitude) of studies are not available in clinical trial databases.

The objective of this work is to implement a novel research platform that processes Clinicaltrials.gov as trial registry, PubMed as medical literature database and a geodatabase in order to render a geographic map of relevant trials or research networks in real-time. As a patient-oriented feature, the search function should support multilingual entry and autosuggestions of diseases to find matching trials. This way, medical terms by laypeople and/or non-English speaking users are mapped to medical concepts, e.g. heart attack or ataque al corazón (Spanish) or Herzinfarkt (German) are mapped to the same concept “myocardial infarction”.

## Methods

### Key features

**Fig. 1:**
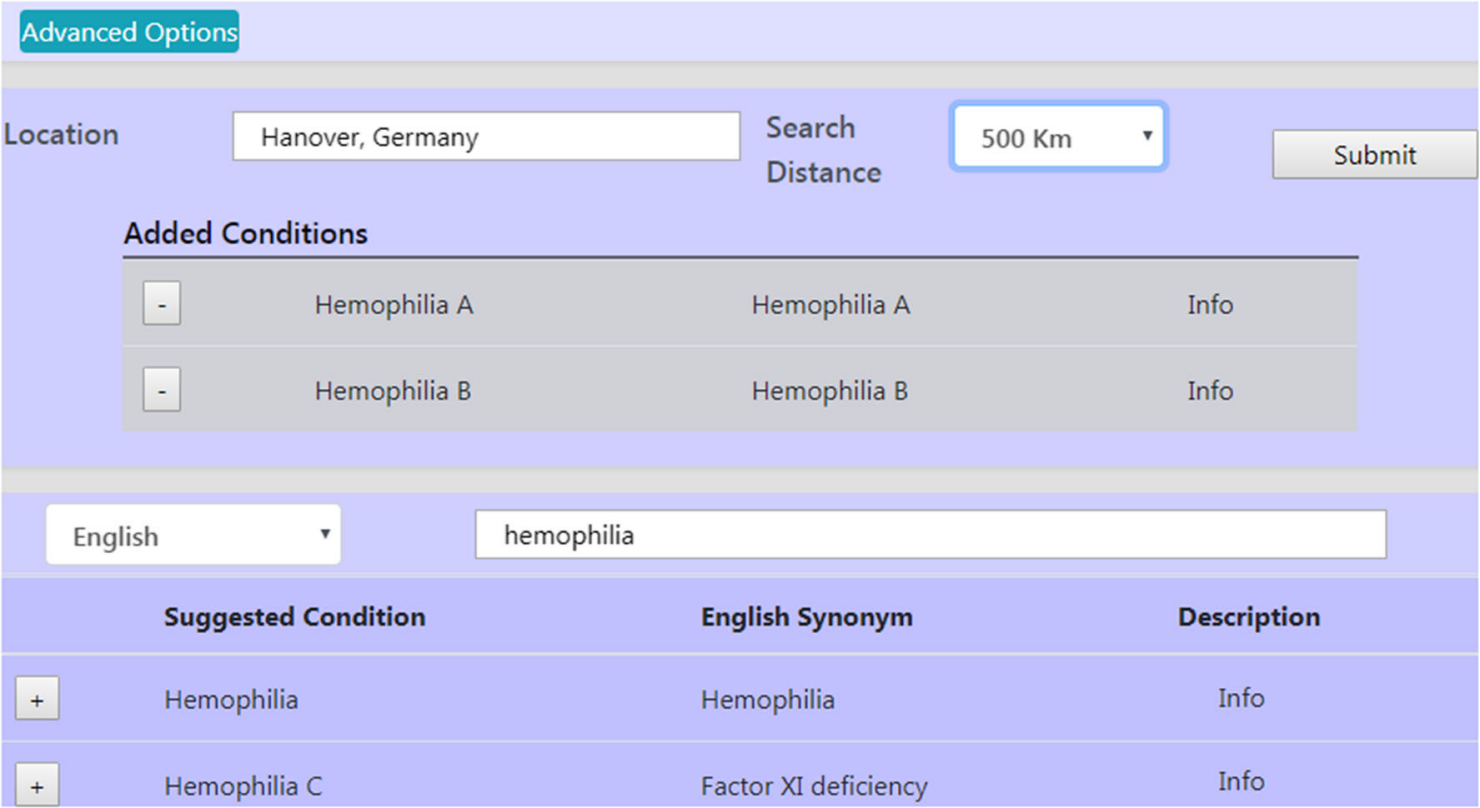
The user interface provides multilingual input and autosuggestion of location and medical conditions. Currently, English, Spanish, French, German and Italian are supported for disease entry. In this example, the user is searching for the condition ‘Hemophilia’ and added ‘Hemophilia A’ and ‘B’, from the list of autosuggested condition terms. Advanced options are used to define the search logics, include PubMed articles, and time ranges

As previous work, key requirements of both use cases were identified as a result of semi-structured interviews with two patient support groups (inflammatory bowel disease and rheumatoid arthritis) and two senior physicians at the local university hospital of Münster in Germany. Four key requirements were identified, which are currently not available on existing international trial registries to the best of our knowledge. KF 1: Generation and visualization of research networks as graphs on a geographic map. Each node of the graph represents a study site of a conducted or conducting trial with facility details on the map. An edge between two nodes represents a collaboration of two sites in one conducted trial. KF 2: Multilingual entry of medical conditions with support of autosuggestion to determine the actual disease concept. Each autosuggested concept is linked with an official description by the Medical Subject Headings (MeSH). KF 3: The search can be filtered for a specific perimeter to find suitable studies within a preferred proximity. KF 4: Further nodes and edges should be shown for any suitable publication found on PubMED (MEDLINE database) in order to boost sensitivity of the research networks and to go beyond clinical trials. That is, if an article is tagged with a MeSH term that is semantically equivalent to the entered medical condition, the affiliations of the lead authors (defined as the first two and last two authors) will be analyzed with the geodatabase and the corresponding locations of the affiliations will be added to the research network. Duplicates will be removed by crosschecking Clinical trial (NCT-IDs) and publication identifiers (PMIDs). Figure 1 illustrates the user interface and the current set of user input options.

### Technical framework

StudyPortal is a Java-based web service implemented as a REST API using Leaflet Library [8] as front-end for mobile-friendly interactive geographic maps. Disease condition terms are indexed with Apache Lucene ™ [9] using concept tables by the Unified Medical Language System (UMLS) [10] in order to provide autosuggestions for the entry of disease conditions. Once an autosuggested term is selected by the user, its Concept Unique Identifier (CUI) will be retrieved from the UMLS table. Each CUI is linked to multilingual MeSH terms or other source vocabularies if available in UMLS. Therefore, the CUI is the basis for finding semantically equivalent terms in ClinicalTrials.gov and PubMed, as both of them use MeSH terms. Trial information is frequently imported from a relational database by ClinicalTrials.gov into StudyPortal’s PostgreSQL database. Imported data contains information on trials, facilities and sponsors including facilities’ locations and ZIP codes, city names and country names. The facilities are mapped against a geospatial location by using the geonames.org database [11] and then visualized via OpenStreetMap – a freely available wiki-like world map [12]. PubMed articles are provided as XML-based MEDLINE data [13]. Articles from MEDLINE contain unstructured affiliation information of the authors. The affiliation texts are parsed through a text-mining algorithm to extract location information. The affiliation is then mapped in descending priority to: 1) a facility name (e.g. University of Leeds) already existing in ClinicalTrials.gov pointing to the specific city (e.g. Leeds, ZIP code: LS184AB); 2) a city of a specific country mentioned in the affiliation text. 3) a city with the highest population (if multiple cities with the same name would be matching and no country information was available).

### Search evaluation

The evaluation on information completeness and visualization correctness for this platform is based on manual cross-checking on other external well-established sources: The WHO International Clinical Trials Registry Platform (ICTRP) [7] for clinical trials and Web of Science for articles. Five trials and five articles were randomly chosen for each of the following three exemplary research-intense disease entities, published between 2014 and September 2018: Breast Cancer, Lung Cancer and Alzheimer Disease (used as search terms on both platforms). Hence, 15 clinical trials and 15 articles were selected and manually tested if they were visualized correctly. A trial (or article) is visualized correctly if all of the study sites (or all article affiliations of lead authors) were correctly localized and visualized within the correct city. To perform this evaluation, we extracted from each tested trial the original trial id and checked for an NCT-ID mapping. For the articles, we extracted the digital object identifier (DOI) and checked for a PMID mapping. If an article or trial had no PMID or NCT-ID mapping it was marked as not retrievable on our platform and thus classified as *not visualized correctly*. To efficiently assess completeness and correctness of visualization, a specific visualization-test platform is accessible via the sub-URL https://studyportal.uni-muenster.de/researcher-network. Here, NCT-IDs of trials and PMIDs of scientific articles can be entered directly and the corresponding research network will be visualized immediately without requiring further user input but using the same core databases and the aforementioned integration procedures. Details of evaluated studies and article are provided in the supplement [14].

## Results

### Implemented key features

**Fig. 2:**
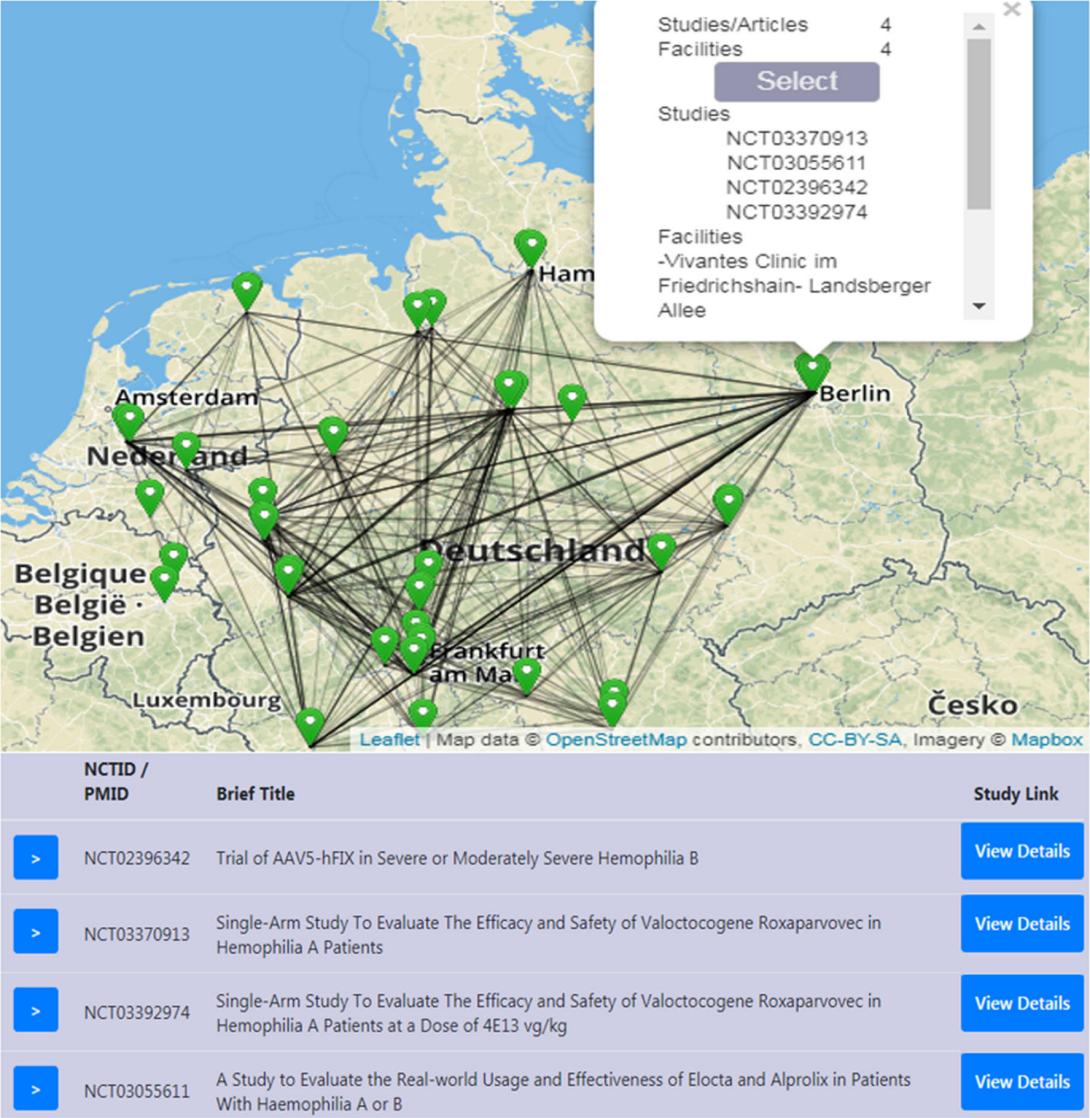
Resulting research network. The user has selected the node at Berlin and can view the corresponding studies, which are linked to the registered trial descriptions (View Details button)

**Fig. 3:**
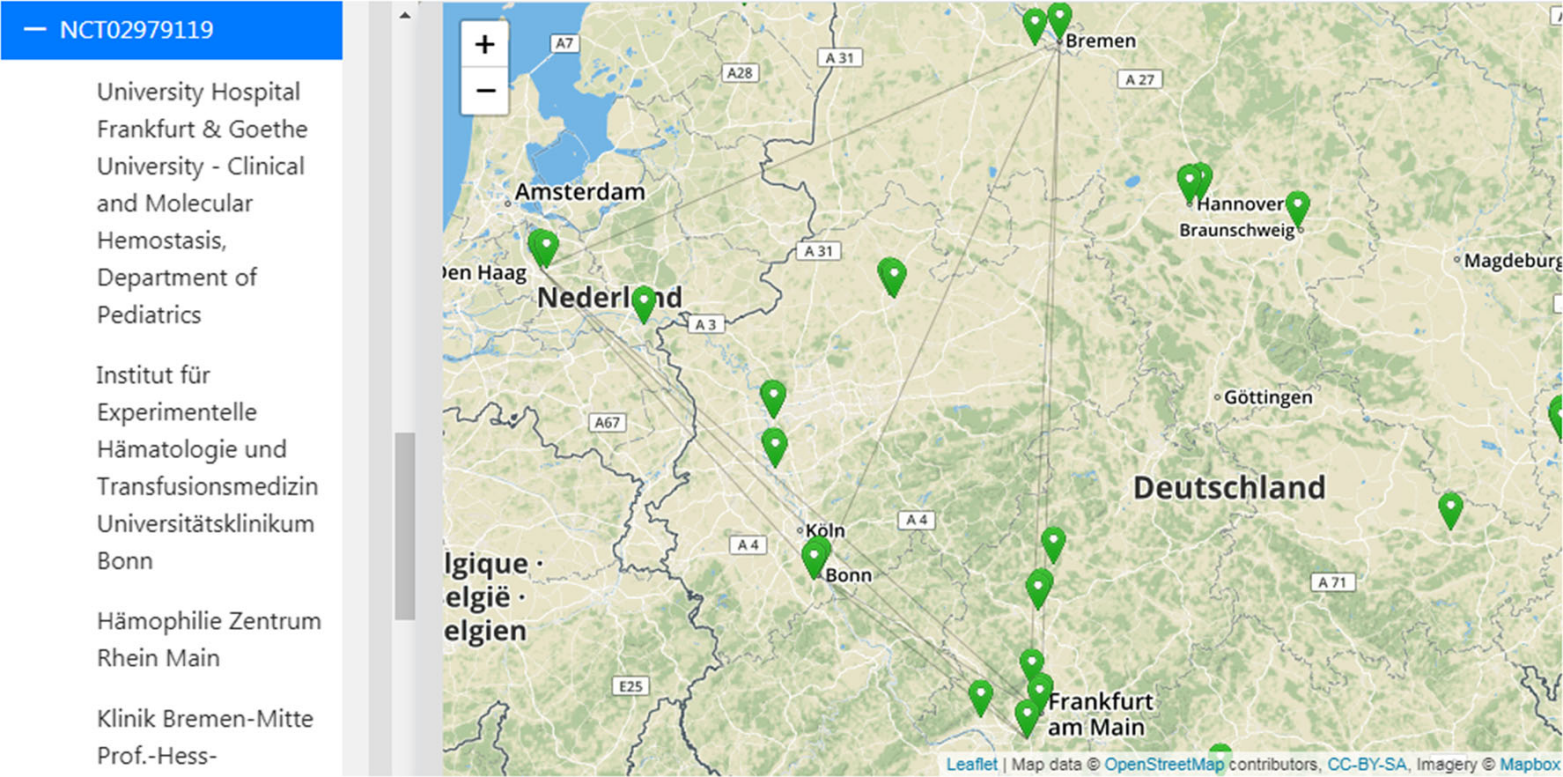
The user has selected one specific study: The PedNet Registry, NCT02979119) and therefore all participating sites of this study are shown as a fully connected network

The platform is accessible on https://studyportal.uni-muenster.de. Import routines to retrieve data from the three core databases ClinicalTrials.gov, MEDLINE and Geonames.org are running on a monthly basis. Figure 2 shows the resulting research network. Each node of the network represent study locations with trials (shown with NCT-identifier) or PubMed articles (with PMIDs) that match the search criteria from Fig. 1. Each study is linked to the original study webpage on ClinicalTrials.gov for detailed study description. Using a navigation view, the user can select a specific study and visualize the corresponding subnetwork as illustrated in Fig. 3.

**Fig. 4:**
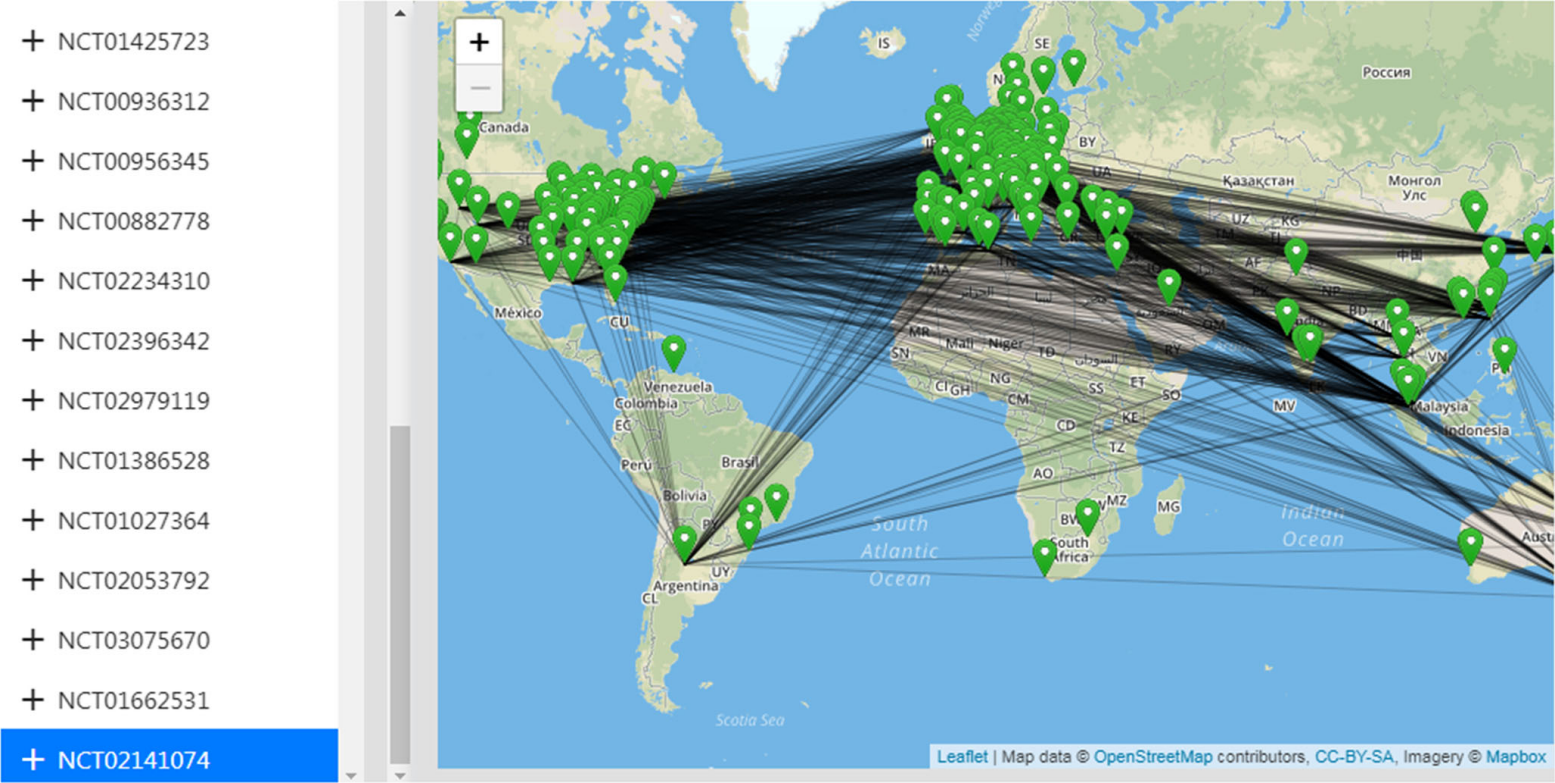
Visualization of the research network with global view for the selected study NCT02141074, which is a phase-3 clinical trial for “Hemophilia B”

While the search radius can be freely selected, the system can directly visualize global study networks: Fig. 4 shows an example of a randomized clinical trial that currently recruits on multiple continents and therefore visualized as a globally connected graph. All result views are fully interactive and support scrolling and zooming in real-time for detailed location views.

### Search evaluation

Expert-based cross-comparison showed that 14 out of 15 (93%) articles and 11 out of 15 (73%) trials were retrievable on StudyPortal. Among of those, all of them (100%) were visualized correctly according to their geographic position. All five misses (1 article +4 trials) were caused by information gaps in ClinicalTrials.gov or MEDLINE. Full tables on evaluation with details on the misses are provided in the supplement [14].

## Discussion

### Implemented key features and future work

The integration of geodatabases, the largest international trial registry, and the largest biomedical literature database was unexploited so far. StudyPortal links these well-maintained but disconnected sources to generate an unprecedented view of studies and research networks on geographic maps. Though all aforementioned key features are implemented and running, the early implementation state cannot encompass further relevant functionalities, which are shortly discussed. Future work will enable extensive filtering of study recruitment status, study phases, interventions, study design and a set of PubMed advanced search filters. These data are already available in structured format and will be planned for next major software release. Moreover, approaches to analyze for hot spot research networks using graph theory-based indicators as centrality measures enables visualization of network evolution over time are subject to current implementation plans as well.

### Search evaluation

Our results indicate that scientific articles could be found and visualized correctly more often (93% vs 73%) than clinical trials. This observation was not surprising as almost all of the tested Web of Science articles were addressing research-intense disease entities and our implementation utilized PubMed, which is one of the largest and most used biomedical databases [15]. The lower coverage for clinical trials is explainable as the US-based ClinicalTrials.gov database is not a dedicated international trial database as the WHO study registry. For instance, three of our 15 test trials were Japanese trials that were not listed on ClinicalTrials.gov. Moreover, many PubMed articles might not be found by our system since these articles were only e-published but not added to the MEDLINE exports of PubMed.

### Limitation

Incomplete study registration is a major limitation of the integrated data sources [3]. The StudyPortal can only visualize study information originating from these sources and therefore cannot close informational gaps. In addition, information on trial registries may not be consistent with original sources, e.g. there is wide variability in the match between published outcomes and those listed in ClinicalTrials.gov [16]. These issues could also mislead patients, which might expect a correct and complete view of the current study landscape. The use of StudyPortal can therefore not replace detailed and critical review of trial outcomes as the purpose of the system is to generate a geospatial overview of the study landscape. Noteworthy, there is a clear trend indicating significant improvements in trial registration, especially for large randomized clinical trials conducted in Europe or US [7]. As preliminary implementation, we had to start from these core databases, since they are maintained by well-established institutions, freely accessible and provide highly structured details on study design and study location. For comparison, the WHO study registry does not provide a free web-service and the EU Clinical Trials Register lacks structured details on study locations compared to ClinicalTrials.gov.

### Conclusion

StudyPortal is the first platform to enable a geospatial overview of biomedical literature and clinical trials. For the majority of tested studies, the presented platform enables an accurate visualization of the study landscape.

## References

[1] Glanville JM, Duffy S, McCool R, Varley D (2014). Searching ClinicalTrials.gov and the international clinical trials registry platform to inform systematic reviews: What are the optimal search approaches?. J. Med. Libr. Assoc..

[2] Cepeda MS, Lobanov V, Berlin JA (2013). From ClinicalTrials.gov trial registry to an analysis-ready database of clinical trial results. Clin. Trials.

[3] Tse T, Fain KM, Zarin DA (2018). How to avoid common problems when using ClinicalTrials.gov in research: 10 issues to consider. BMJ.

[4] de Angelis CD, Drazen JM, Frizelle FA, Haug C, Hoey J, Horton R, Kotzin S, Laine C, Marusic A, Overbeke AJP, Schroeder TV, Sox HC, van der Weyden MB (2005). Is this clinical trial fully registered?. Ann. Intern. Med..

[5] Food and Drug Administration. Food and Drug Administration Amendments Act of 2007: public law 110–85 2007.: Available at http://www.gpo.gov/fdsys/pkg/PLAW-110publ85/pdf/PLAW-110publ85.pdf. Accessed Aug 21, 2018. [cited 2018 Dec 8]. Available from: URL: http://www.gpo.gov/fdsys/pkg/PLAW-110publ85/pdf/PLAW-110publ85.pdf.

[6] Roberto A, Radrezza S, Mosconi P (2018). Transparency in ovarian cancer clinical trial results: ClinicalTrials.gov versus PubMed, Embase and Google scholar. J. Ovarian Res..

[7] Knelangen M, Hausner E, Metzendorf M-I, Sturtz S, Waffenschmidt S (2018). Trial registry searches for randomized controlled trials of new drugs required registry-specific adaptation to achieve adequate sensitivity. J. Clin. Epidemiol..

[8] Derrough J (2013). Instant Interactive Map Designs with Leaflet JavaScript Library How-to: Packt Publishing Ltd.

[9] Białecki, A., Muir, R., Ingersoll, G., editors. Apache lucene 4; In SIGIR 2012 workshop on open source information retrieval (p.17), 2012.

[10] Bodenreider O (2004). The unified medical language system (UMLS): Integrating biomedical terminology. Nucleic Acids Res..

[11] Ahlers D (2013). Assessment of the accuracy of GeoNames gazetteer data. Proceedings of the 7th workshop on geographic information retrieval.

[12] Haklay M, Weber P (2008). OpenStreetMap: User-generated street maps. IEEE Pervasive Comput..

[13] National Library of Medicine. Download MEDLINE/PubMed Data. [cited 2018 Oct 21]. Available from: URL: https://www.nlm.nih.gov/databases/download/pubmed_medline.html.

[14] Evaluation, Varghese, J. Table for trials and articles that were tested on Studyportal. [cited 2018 Oct 21]. Available from: URL: https://uni-muenster.sciebo.de/s/JU1k89MGxSLpZ72.

[15] Falagas ME, Pitsouni EI, Malietzis GA, Pappas G (2008). Comparison of PubMed, Scopus, web of science, and Google scholar: Strengths and weaknesses. FASEB J..

[16] Adam GP, Springs S, Trikalinos T, Williams JW, Eaton JL, von Isenburg M, Gierisch JM, Wilson LM, Robinson KA, Viswanathan M, Middleton JC, Forman-Hoffman VL, Berliner E, Kaplan RM (2018). Does information from ClinicalTrials.gov increase transparency and reduce bias? Results from a five-report case series. Syst. Rev..

